# Biodegradable and biocompatible high elastic chitosan scaffold is cell-friendly both *in vitro* and *in vivo*

**DOI:** 10.18632/oncotarget.14709

**Published:** 2017-01-17

**Authors:** Yichuan Pang, An Qin, Xianfeng Lin, Lin Yang, Qiang Wang, Zhengke Wang, Zhi Shan, Shengyun Li, Jiying Wang, Shunwu Fan, Qiaoling Hu

**Affiliations:** ^1^ Department of Polymer Science and Engineering, MOE Key Laboratory of Macromolecular Synthesis and Functionalization, Zhejiang University, Hangzhou, China; ^2^ Department of Orthopedic Surgery, Shanghai Key Laboratory of Orthopedic Implants, Shanghai Ninth People's Hospital, Shanghai Jiao Tong University School of Medicine, Shanghai, China; ^3^ Department of Orthopaedics, Sir Shaw Run Run Hospital, School of Medicine, Zhejiang University, Hangzhou, China

**Keywords:** chitosan, elastic scaffold, osteogenesis, tissue engineering

## Abstract

Biodegradable and biocompatible macromolecule chitosan has been favored for a variety of clinical applications. We reported herein the fabrication of a novel chitosan scaffold with high elasticity. This scaffold can be easily compressed and thus enable the insertion of such scaffold into surgical lesions during minimal invasive surgeries. In addition, this novel scaffold can restore its shape when released. We evidenced that this high elastic scaffold has better biocompatibility than traditional chitosan scaffold. Therefore, this new chitosan material might lead to the manufacture of a variety of novel biodegradable biomedical materials and devices.

## INTRODUCTION

Bone defect repair is still a challenge for orthopaedic surgeons. Autologous bone graft is the golden standard to treat bone defect. However, autologous bone graft is associated with complications including limited bone sources, donor site pain and possible donor site infection. As a replacement, allogeneic or xenogeneic bone was alternatively used. But potential risks such as disease transmission and immune rejection are still the problem [1–3].

To overcome these problems, extensive researches focus on generating novel biomaterials for bone grafting. Metal (titanium [4], magnesium [5]), inorganic non-metal (bio-glass [6]), organic (PEEK [7], PLA/PGA [8], sodium alginate [9], chitosan [10], hyaluronic acid [11]) and organic-inorganic combination (bone cement [12], HA/PLA) were greatly developed for bone defect repair. Till now, inorganic materials were still commonly used due to their stable chemical properties and excellent mechanical strength. But these materials are associated with long-term adjacent bone loss because of the stress shielding effect [12]. Organic and organic-inorganic combined materials are preferred since they have potential to be modified and processed with different function (like bioactive, biodegradable) or forms (porous, ordered). Especially, biodegradable materials are extensively studied. For instance, biodegradable synthesized polymer (PLA, PGA) had been reported as bone repair scaffolds either fabricated by 3D printing or template method to regenerate bone tissue [13].

Chitosan is another biodegradable material that has been widely used in clinics. It is a natural macromolecule produced by deacetylation of chitin that derived from the cuticles of crustaceans such as shrimp and crab shells, or from insects (maggot, silkworm chrysalis) and other microorganisms (bacteria, fungus and mycete) [14]. Importantly, chitosan has similar structure and composition to glycosaminoglycans (GAGs) and thus elicits minimal immune response when implanted in human body. Therefore, chitosan is a non-toxic, biodegradable and biocompatible macromolecule that has gained interests for biomedical applications [10, 15–21]. Chitosan has been fabricated as nanoparticles for drug (gene) delivery [22–30]. Hydrogel made from chitosan has also been used to absorb metal ions or other chemicals for anti-bacterial and anti-tumor applications [31, 32]. In addition, chitosan hydrogel can be hybrided with other composition such as hydroxyapaptite for tissue engineering [33–38].

When come to bone repair scaffold, the elastic property is a neglected property that is important for some of the bone repair conditions. For instance, high elastic scaffold that can be easily compressed and delivered through the endoscope tube will facilitate mini-invasive surgical operations for organ repair including non-loaded bone repair. Compressed scaffold that can restore its shape after delivered into the surgical lesion will further minimize surgical incision during cosmetic operations. However, most of natural and synthesized macromolecules are non-elastic due to their hydrogen bonding and high crystallinity.

**Scheme 1:**
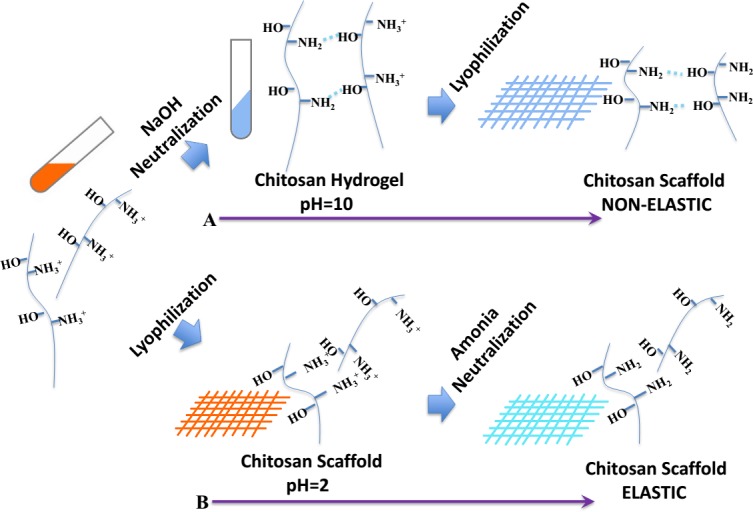
Schematic illustration of the farbrication procedure by (A) NaOH neutralization and lyophilization that result in non-elastic scaffold or (B) lyophilization followed by NH3 neutralization that result in elastic scaffold

Chitosan is a suitable bone regeneration material above all. But as the same with the most natural macromolecules, chitosan has a rigid chain connected by sugar rings and mass of hydrogen bonding that result in no elasticity in chitosan. Inspired by these clinical needs, we aim to fabricate pure chitosan scaffold with high and durable elastic property. To achieve this, as shown in Scheme 1, we used a new processing technique to fabricate the chitosan scaffold with high elasticity in liquid by limitation the hydrogen bonding.

## RESULTS

**Figure 1:**
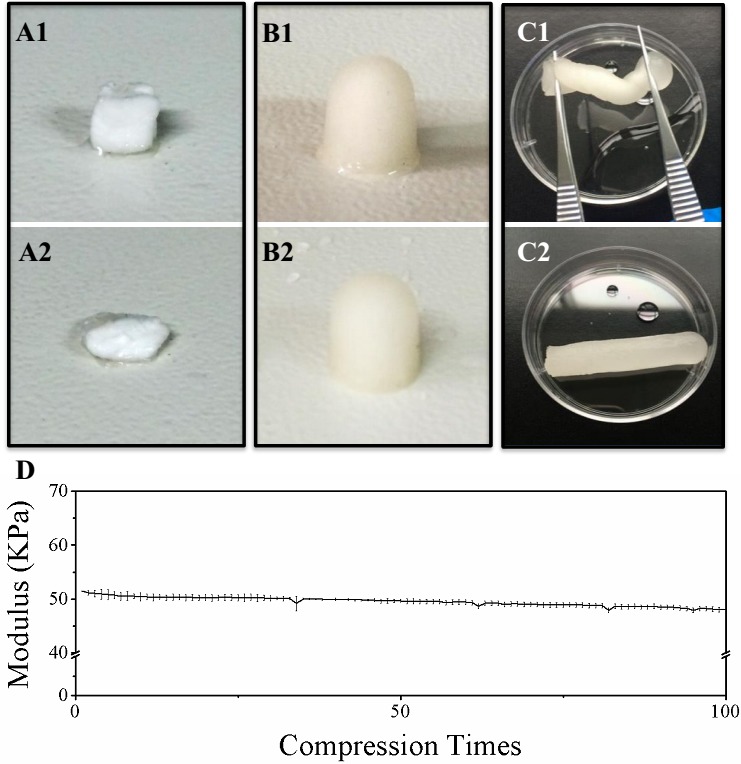
Evaluation of chitosan scaffold elasticity **A**. Morphological change of chitosan scaffold neutralized by NaOH (CSS) before and after 500g pressure. **B**. Morphological change of chitosan scaffold neutralized by NH3 (CSA) before and after 500g pressure. **C**. CSA twisted and released. **D**. The stability of CSS elasticity during repeated compression.

Ammonia was finally chosen to neutralize chitosan, which is different from traditional way of using sodium hydroxide to neutralize chitosan. Figure 1A1&1A2 showed the shape of chitosan scaffold made by traditional way of sodium hydroxide (CSS) before and after the press by a 500g weight. The scaffold was easily smashed after the press. In contrast, the chitosan scaffold neutralized by ammonia (CSA) can restore its shape after the press by a 500g weight (Figure 1B1&1B2). Moreover, the novel scaffold can be twisted (> 360°/cm) and immediately restore its shape when released (Figure 1C1&1C2). Furthermore, the stability of the elastic scaffold was tested by given repeated (100 times) press with deformation beyond 80%. Only about 2% of elastic modulus lost (Figure 1D). Together, these data suggested that this novel chitosan scaffold is of high elastic property. When come to the compress modulus, the elastic scaffold still keeps other mechanical properties similar as the traditional one (Figure S1A, S1B&S1C).

**Figure 2:**
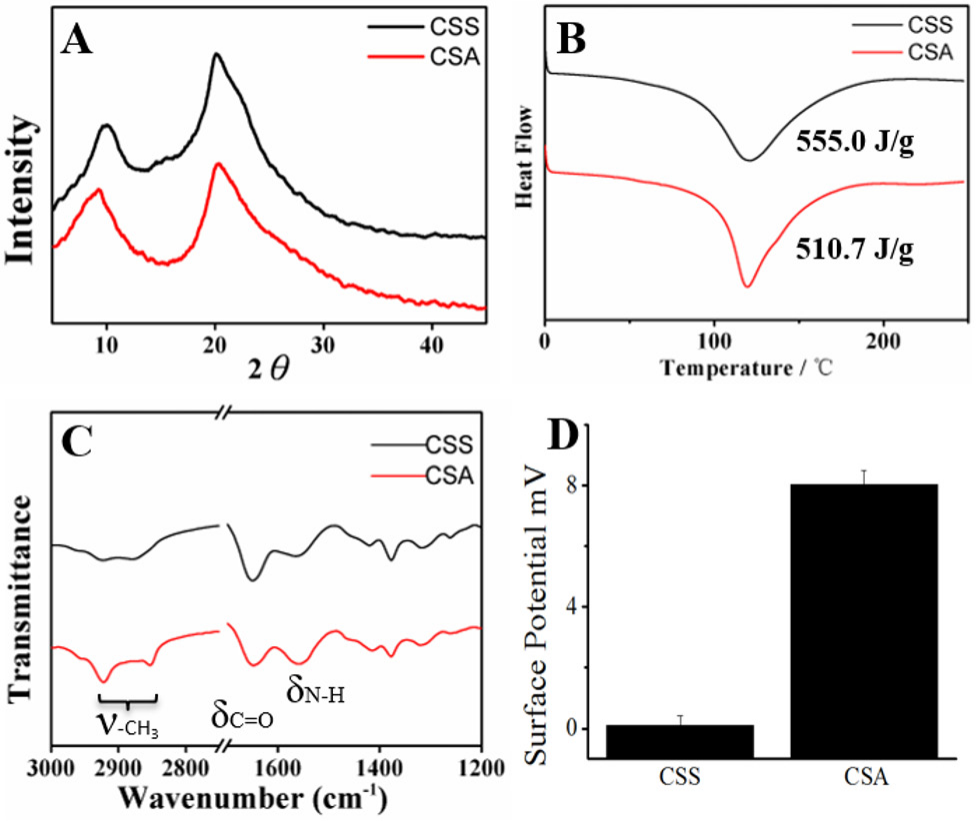
Crystallization and group state analysis of scaffolds **A**. XRD of two scaffolds; **B**. DSC test of two scaffolds; **C**. ATR-FTIR of two materials; **D**. Surface potential of CSS and CSA membranes.

Scaffolds structure was measured by scanning electron microscope (SEM) and we find that the CSS possess oriented, smaller and pipe like structure while the CSA own random but suitable size pores for cells (Figure S2A&S2B). These two scaffolds has similar porosity (91.39±0.81% for CSS, while 87.97±3.28% for CSA) but different pore diameter (2.3±0.5μm for CSS, while50±4μm for CSA) (Figure S2C&S2D). The material properties of CSA and CSS in conformation and group states were further characterized by X-Ray diffraction (XRD), differential scanning calorimetry(DSC), attenuated total refraction Fourier transform infrared spectroscopy (ATR-FTIR) and zeta potential test respectively. Both XRD and DSC analysis revealed that crystalline area is almost the same in both scaffolds (Figure 2A&2B). However, the crystalline area of two kinds of scaffolds share the same crystal form in other works the same diffraction peaks and the similar degree of crystallinity. The wide absorption peak in 3400-3500 cm^−1^ in infrared spectrum related to -N-H and -O-H stretching vibrations of chitosan (3307 cm^−1^ refers to amide N-H stretching, 3366 cm^−1^ refers to O-H stretching) (Figure S2E) [39, 40]. In addition, the peak observed at 2926 cm^−1^ and 2878 cm^−1^ can be assigned to the stretching vibrations of -CH_3_ which was stronger in CSA. Moreover, the amide-| C = O stretching vibration contribute to an absorption in 1652 cm^−1^, which is similar between the two scaffolds. Finally, the absorption band observed in 1564 cm^−1^ given by amide-II, N-H bending was stronger in CSA than CSS. Together, these data suggested that stronger hydrogen bonding bridge between -OH and -NH3; -OH and -COCH_3_ in the CSS, reflected by weaker vibration of groups in CSS scaffold (Figure 2C). Interestingly, we noticed the CSA membrane has higher surface potential than the CSS membrane (Figure 2D) (the CSA was about 8.01±0.48 mV while the CSS was about 0.09±0.32 mV.) That means the CSA membrane was stronger in capturing hydrogen ion in neutral environment (PBS buffer). There is more “free amino groups” in CSA scaffold. In agreement with this, the CSA scaffold was tougher in ethanol (Figure S1), suggesting the free un-hydrogen bonding group can catch hydrogen ion in water and stretch in some degree but cannot do the same thing in ethanol. The FTIR and surface potential suggest that there are increasing number of hydrogen bonding in the amorphous areas in CSS while two kinds of scaffold share the same crystal type and ratio. In conclusion, the novel chitosan scaffold's elasticity was obtained by less hydrogen bonding in amorphous and strength was acquired by crystalline region.

**Figure 3:**
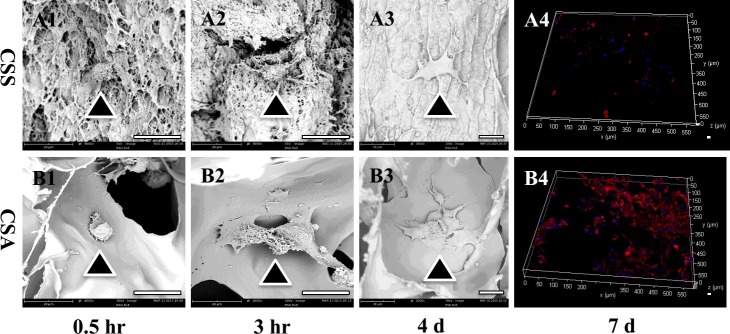
Biocompatiblity of both chitosan scaffolds *in vitro* (**A1**&**B1**) Cell attachment on the CSA or CSS scaffold for 0.5h. (**A2**&**B2**) Cell attachment on the CSA or CSS scaffold for 3hr. (**A3**&**B3**) Cell proliferation on the CSA or CSS scaffold for 4 days. (**A4**&**B4**) Cell proliferation on the CSA or CSS scaffold for 7 days. The scale bar is 20 μm for A1-A3 & B1-B3; 100 μm for A4 &B4. Scale bar is 20 μm.

The biocompatibility of both chitosan-derived materials was initially evaluated *in vitro*. Only about 20% of the MC3T3-E1 cells attached onto the CSS membrane while about 60% of the MC3T3-E1 cells attached onto the CSA membrane within 1 hour. The cells just stretching out their parapodium on CSS while well spreading on CSA at 0.5h (Figure 3A1&3B1, Figure S3A&S3B). After 3 hours’ incubation, the cells on the CSA spread way better than the cells on the CSS (Figure 3A2&3B2). More than 60% of the MC3T3-E1 cells attached onto the CSS membrane after 4 hours while more than 90% cells attached onto the CSA membrane at this time point (Figure S3A&S3B).

The proliferation of bone marrow derived mesenchymal stem cells (BMSCs) and MC3T3-E1 cells on both scaffolds were further investigated. There is hardly any cell proliferation observed on the CSS membrane. In contrast, MC3T3-E1 cells on the CSA membrane proliferate more vigorously (Figure S3C&S3D). In agreement with this, BMSCs proliferated on the CSA membrane but not on the CSS membrane within 7 days (Figure S3E&S3F). Scanning electron microscope images further confirmed that BMSCs proliferated better on the CSA than CSS after 4 days (Figure 3A3&3B3). There are numerous BMSCs on the CSA scaffold while the number of BMSCs is very limited on CSS after 7 days (Figure 3A4&3B4).

The differentiation of BMSCs on CSA scaffolds was further examined. As shown in Figure S4, increasing alkaline phosphatase (ALP) positive nodules can be noticed on the CSA membranes seeded with BMSCs after osteogenesis induction for 4,7 and 14 days respectively. To further confirm the osteogenesis of BMSCs on the CSA membrane, relative osteogenic gene expression was evaluated. As shown in Figure S5, osteoblastic collagen type I, collagen type III, ALP and RunX2 significantly increased during osteogenesis. Collectively, these data suggested CSA has better performance than CSS in supporting cell attachment, proliferation and differentiation *in vitro*.

**Figure 4:**
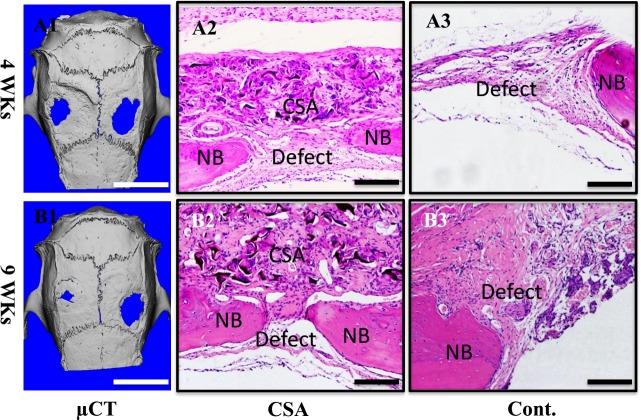
Biocompatiblity of CSA scaffolds *in vivo* **A**)implant for 4 weeks, **B**)implant for 9 weeks; **A1**&**B1**) microCT images of SD rat cranium, left-CSA, right-control. **A2**-**A3**, **B2**-**B3**) HE stain. **A2**-**A3**) are 4 weeks, **B2**-**B3**) are 9 weeks. **A2**&**B2**) CSA groups, **A3**&**B3**) control groups. Scale bar is 5 mm in **A1**&**B1**, 100 μm in **A2, A3, B2**&**B3**.

The biocompatibility of this high elastic scaffold was then examined *in vivo*. Due to its high elasticity, CSA scaffold was easily sutured onto the calvarial bone defect. MicroCT revealed that the bone defect in CSA scaffold group healed better than the control group at 4 weeks. The healing process was further accelerated in the CSA group at 9 weeks, where almost the whole bone defect has been repaired (Figure 5A & 5B). The ratio of bone volume and tissue volume (BV/TV) in high elastic chitosan group was 0.73±0.02 while just 0.57±0.04 in the control group at 4 weeks. The CSA group increased to 0.82±0.05 at 9 weeks while 0.65±0.04 in control group (Figure S6). Histological assessment revealed that the chitosan scaffold exhibited good compatibility with tissue. A little but reasonable inflammatory reaction was observed in CSA group. It promoted bone defect healing as demonstrated by narrower bone defect on the H&E images and masson staining images at both 4wks and 9wks (Figure 4A2-4A3, 4B2-4B3 & Figure S6). Collectivelly, the high elastic chitosan scaffold has good biocompatibility *in vivo*.

## DISCUSSION

To explain why NaOH neutralization results in non-elastic scaffold, we reasoned that when deprotonation and neutralization was processed before lyophilization, the hydrogen bonding can easily form in the scaffold. To avoid this, we chose to lyophilization before neutralization by ammonia to persist the formation of hydrogen bonding and thus leads to the existence of “free amino-groups” that contribute to the elastic property in water. The schematic explains the process how it going. When the chitosan was dissolved, the -NH2 group was protonated to become NH3+. Subsequent lyophilization of the solution generated the acid sponge, with unformed hydrogen bonding between NH3+ groups and -OH groups. And more free volume of chains gives the novel scaffold high elasticity. Benefited from these factors, the CSA display a very stable elastic behavior in water. So the scaffold can be delivered through endoscopic tubes and restore its original shape to meet clinical needs. Moreover, the XRD and DSC also confirm the crystalline region was not break by the new method. So the CSA scaffold was endowed better mechanical properties whether dry or in water.

Coincidentally, the *in vitro* test of CCK-8 also confirm the CSA scaffold was a suitable medium to osteogenesis precursor cells. It raises 69.8% of the cells at the beginning while just 19% for CSS. When come to this, the free amino group may contribute most. It gives the material flexible property and positive charge to attractive cell to attach on it. What's more, the cell state on the CSA scaffold was better. They proliferate 3 times in 48h and 2 times in 24h compared with 12h in CSA scaffold. The same situation was observed in 4d and 7d (2.1times and 3.1 times compared with 1d) (See Figure S3). On the contrary, the cells never proliferate on CSS scaffold and we believe the residual alkali may lead to this. Moreover, the RNA expression of osteogenesis related genes was up regulated on CSA scaffold stay the same on CSS scaffold (see Figure S5). This also can be confirmed by ALP stain in Figure S4. The *in vivo* test is compatible with the *in vitro* test. These result all point out that the CSA scaffold was an excellent tissue engineering scaffold for bone regeneration.

## CONCLUSION

In summary, we fabricate a novel high elastic chitosan scaffold by using ammonia to inhibit hydrogen bonding. This scaffold can be compressed and bounce back to its original shape immediately. Both *in vitro* and *in vivo* study proved the high elastic scaffold is more friendly to cells and efficient to bone defect repairmen compared with traditional chitosan scaffolds. Therefore, we believe that the novel scaffold possesses the potential to meet clinical needs of elastic, biodegradable and biocompatible scaffolds.

## MATERIALS AND METHODS

### Elastic chitosan sponge fabrication

Chitin was pursued from Zhejiang Golden-Shell Biochemical Co.,Ltd. After being washed in dd-H_2_O and dried in 60°C, chitin was smashed and put it in NaOH solution (50wt%) at 70°C for 4 h, followed by neutralization in dd-H_2_O. This procedure was repeated for 5 times to make sure the deacetylation is >95% (Chitosan with high degree of deacetylation—CS/HDD). Two grams of CS/HDD powder were dissolved in 90ml 1%vt acetic acid solution, followed by adding 120ml methanol to the system and filtrated. Then 0.7 ml acetic anhydride was dropped into the solution and the system was stirred for 15 minutes. Aftet standing for 2 hours, chitosan was deposited by sodium hydroxide solution, neutralized by washing and dried. The chitosan with a uniform distribution of acetyl amino groups and amino group in main chain (CS/UD) was achieved.

The elastic chitosan sponge was prepared by the following steps: Frist, 4 grams of chitosan was dissolved in 100 ml 1%vt acetic acid solution to get 4% chitosan solution. Second, chitosan solution was filled into the mould, standing to defoam and frozen in -20 °C over night. Then, the freezing solution was lyophilized and neutralized using ammonia by filling it to the chamber.

In addition, traditional chitosan scaffold was prepared as a control group. Chitosan was filled into the mould than then carefully dipped into 5% sodium hydroxide solution for neutralization to form gel, washed by ddH_2_O to neutral and lyophilized. The process of gelatinization need more than 30 minutes and even more times depending on the size of mould.

### Scaffold characterization

The molecular weight of chitosan and degree of deacetylation was measured according to the papers published before. For scanning electron microscope (SEM), the scaffold was fixed and dehydrated by ethanol. Then it was lyophilized and coated with a thin gold layer. The mechanical measurement was carried out by Instron mechanical tester. Attenuated Total Refraction Fourier transform infrared spectroscopy (ATR-FTIR, Themo Fisher scientific LLC) and X-ray diffraction (XRD) were implemented by flattening the scaffolds into pieces then put them in 40°C for 2 h and send into the chambers of instruments. For differential scanning calorimetry (DSC) analysis of the thermal behavior of scaffold, we provided a temperature of 0-250°C by a ratio of 2 degree per minute. For the surface potential test, two films fabricated by the same way as chitosan scaffolds were used and tested. The porosity and pore diameter of the scaffolds were analyzed by mercury intrusion porosimetry.

### Cell culture

New Zealand rabbit (8 weeks old) was anesthetized by pentobarbital sodium. The bone marrow was extracted from the iliac crest by medulla-puncture needle and separated by density gradient centrifugation with Ficoll. The obtained cells were seeded in T75 flask, supplemented with DMEM containing 10% FBS and 1% penicillin/streptomycin in 5% CO2 and humid incubator. The second passage of BMSCs were used for the following experiment. MC3T3 cells were cultured with DMEM containing 10% FBS and 1% penicillin/streptomycin.

### Cell viability assay

Both MC3T3 and BMSCs were used for cell viability assay. Scaffolds (6mm in diameter 1mm in thickness were prepared, sterilized and seeded d with 5×10^5^ cells/well in the 24 well plate. The scaffold was washed with PBS for 3 times before adding cell count kit-8 (CCK-8) assay solution at the indicated time point. It was incubated for 2 hours before measuring the absorbance of culture medium.

### Confocal microscopy and SEM imaging

After being cultured until the indicated time point, the scaffolds were fixed with 4% formaldehyde for 20 minutes, followed by 0.1% Triton X-100 permeabilization for 10 minutes. Then scaffolds were washed by PBS for 3 times and treated with 1% BSA for 1hour. The scaffolds were then incubated with Phalloidin-rhodamine and DAPI for 1hour at 4°C before washing with PBS. The samples were then imaged under Laca Zeiss LSM.

For SEM imaging, the scaffold was fixed and washed with PBS. Then it was dehydrated by ethanol with a gradient of 50%, 75%, 95% and 100% for 1 hour respectively. After being treated with tertiary butanol, the scaffold was lyophilized for imaging.

### BMSCs differentiation and alkaline phosphate activity staining

Osteogenesis medium was prepared by adding sodium glycerophosphate (1M), Vitamin C (10mM) and Hexadecadrol (1mM) to the DMEM medium supplemented with 10% FBS and 1%penicillin/streptomycin. The Scaffold (Ф6mm*1mm) was seeded with 5×10^5^ BMSCs for osteogenesis. The scaffolds were fixed with 4% paraformaldehyde at day 4,7 and 14. ALP stain Kit was used for imaging the ALP positive nodules under microscope.

### RNA extraction and quantitative PCR assay

The RNA was isolated from cells according to the TRIzol and choloform methods. The extracted RNA was synthesized into cDNA for realtime assay using SYBR Green. The expression of collagen 1, collagen 3, ALP and RunX 2 in BMSCs was evaluated. β-actin was chosen as the housekeeping gene.

### *In vivo* osteogenesis assay

Twelve male SpragueeDawley rats (12-week-old) with a weight of 250±20 g were used under the animal ethics approved by the Zhejiang University Ethics Committee. The rats were anesthetized by an intraperitoneal injection of pentobarbital (3.5 mg/100 g). Then the cranium was shaved and sterilized. A bone defect of 6 mm in diameter was created on parietal bone in each side of the calvaria bone. One defect was treated with covered with the CSA scaffold and the other side was left blank as control. The periosteum and skin were closed. The calvaria were harvested for microCT, HE stain and Masson staining at 4 weeks and 9 weeks post-operation.

### Statistical analysis

Biological data of this study was analyzed using ANOVA. Results are reported as mean±SD deviation. And the ** represents for the significant level p<0.01, while * represents for *p*<0.05.

## SUPPLEMENTARY MATERIALS FIGURES


