# Supplementary material for: Biodegradable and biocompatible high elastic chitosan scaffold is cell-friendly both in vitro and in vivo
Source: Oncotarget. 2017 Jan 17;8(22):35583–91. doi: 10.18632/oncotarget.14709 (PMC5482600; doi:10.18632/oncotarget.14709)
Supplement: Supplementary file 1 [file oncotarget-08-35583-s001.pdf]

Biodegradable and biocompatible high elastic chitosan scaffold is cell-friendly both *in vitro* and *in vivo*

Supplementary Material

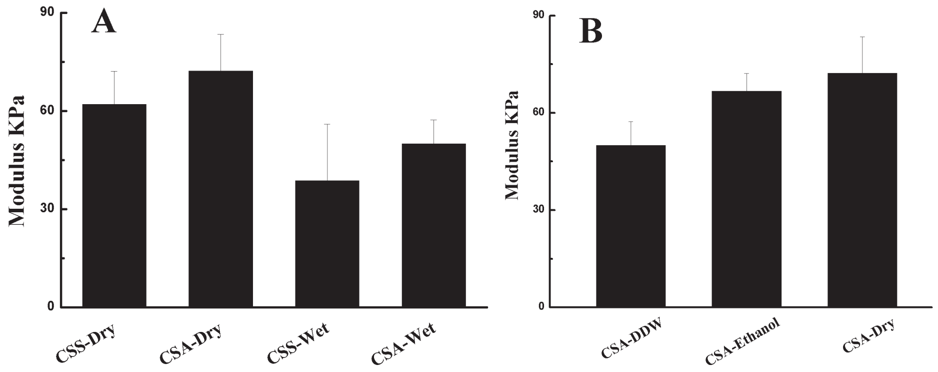

Supplementary Figure S1: Compress Modulus of CSS and CSA. A) Two kinds of scaffolds compress at dry and wet; B) Compress the CSA scaffold at different conditions..

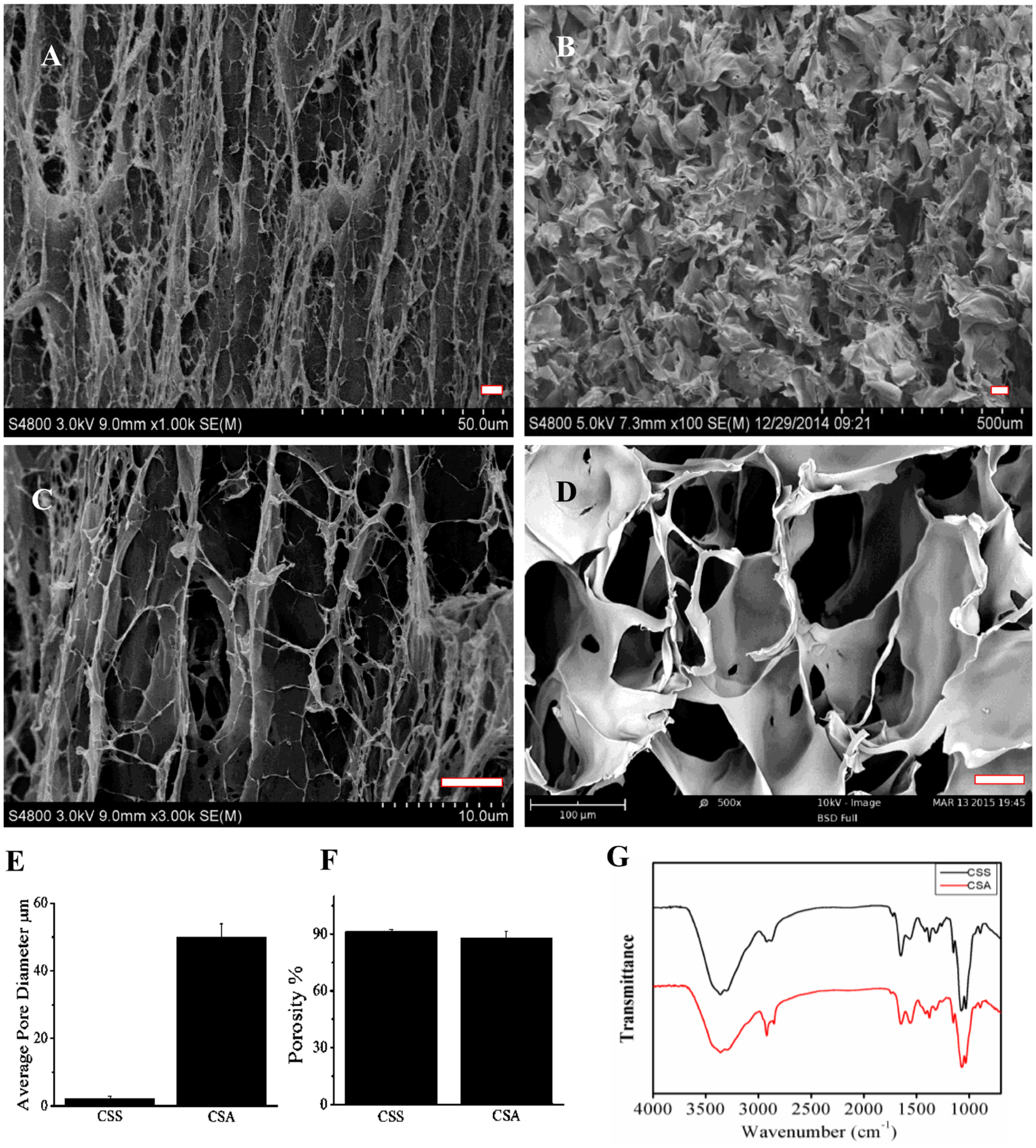

Supplementary Figure S2: Characters of CSS and CSA scaffold of A&C) SEM imagings of CSS and B&D) of CSA; E) average pore diameters; F) porosity of the two scaffolds. G) ATR-FTIR spectrum of CSS and CSA scaffolds. Scale bar for A&C is 5 µm, B&D is 50 µm..

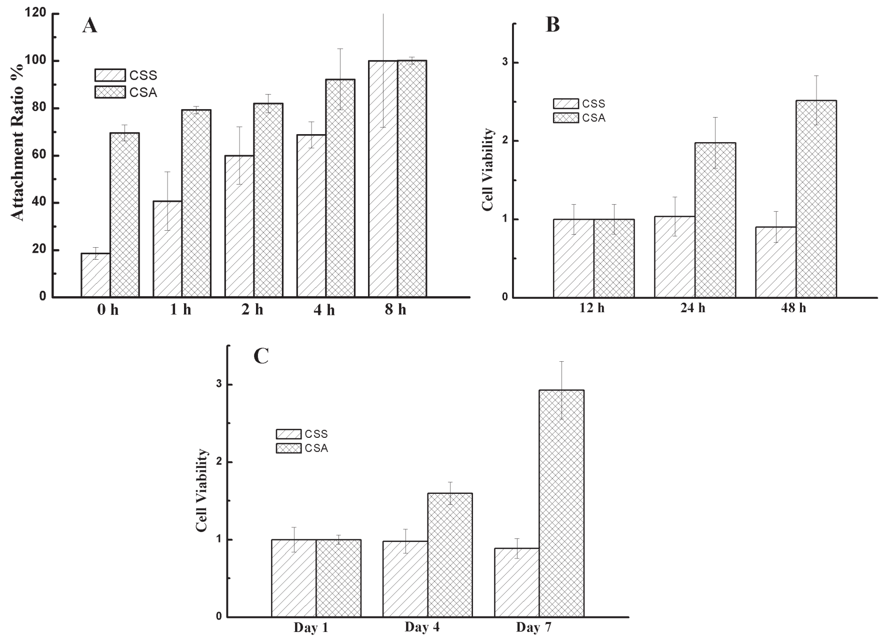

Supplementary Figure S3: Cell viability assay of CSS and CSA. A) MC3T3 cell's attachment; B&C) Cell proliferation of BMSC on CSS and CSA for different times.

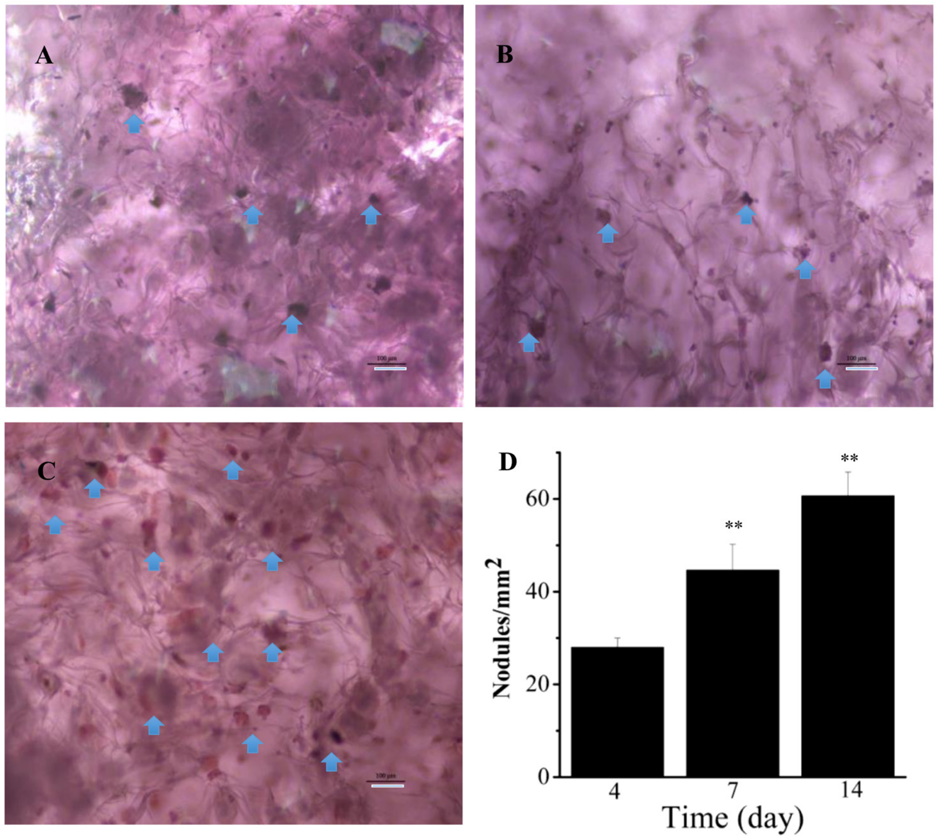

Supplementary Figure S4: Alkaline Phosphate Activity Staining of CSA scaffold. A, B, C) was ALP stain imagings of differentiation for 4, 7, 14 days and D) was the statistics of A,B and C. Scale bar is 100 μm.

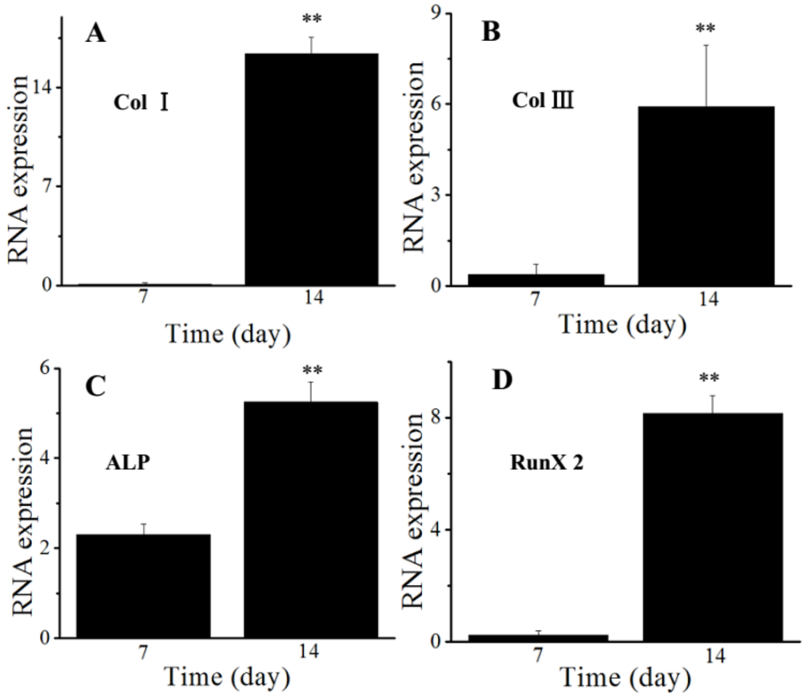

Supplementary Figure S5: Corresponding RNA expression of osteogenesis. A) expression of collegan type one; B) expression of collegan type three; C) ALP express and D) RunX2 express.

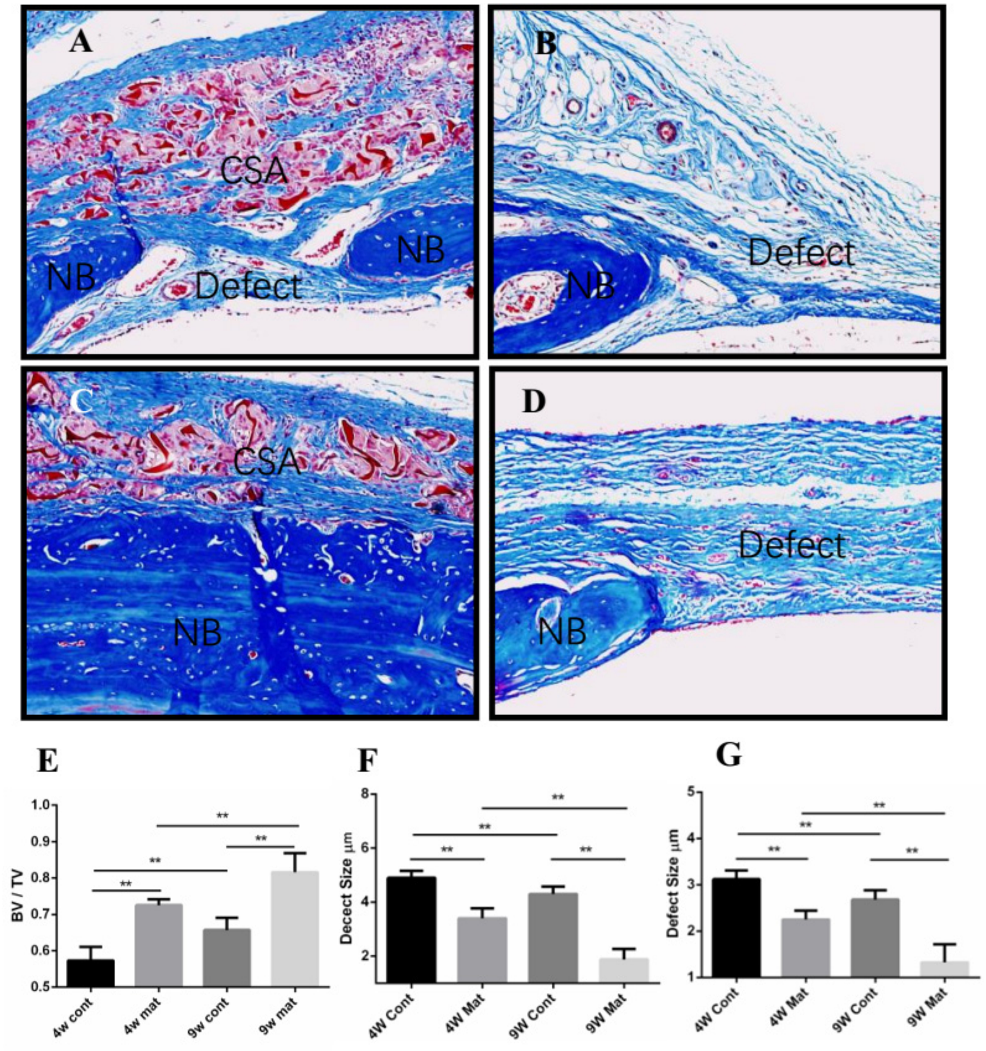

Supplementary Figure S6: Osteogensis assary in vivo. A-D) Masson stain of bone defect repaired tissue. A) scaffold group for 4 weeks; B) control group for 4weeks; C) scaffold group for 9 weeks; D) control group for 9 weeks; E) BV/TV of bone defect area. F&G) maximum and minimum diameter of bone defect.
